# Amnesic and Ataxic Aphasia, Etc.

**Published:** 1872-06

**Authors:** T. M. B. Cross

**Affiliations:** Assistant to the Chair of Diseases of the Mind and Nervous System, Bellevue Medical College, New York, etc.


*Amnesic and Ataxic Aphasia, with Agraphia and Temporary Right Hemiplegia, the result of Embolism of the Left Middle Cerebral Artery.* By T. M. B. Cross, M.D., Assistant to the Chair of Diseases of the Mind and Nervous System, Bellevue Medical College, New York, etc.

Captain D., thirty-two years of age, was born in England, is married and the father of two children, one of whom is at present living. A strong, robust man, of medium size, fine constitution, and sanguine temperament, he has always enjoyed the best of health, and has never suffered in his life from any disease of moment which he can at present recall; nor has he ever been afflicted with rheumatism, gout, or syphilis, so far as he is aware. In tracing his family line down to the present time, there is no history of diseases of the nervous system among any of its members, either on the maternal or the paternal side, until we come to his brother, who is an epileptic, and then again to one of his children, who died in infancy of epileptiform convulsions. Even in regard to other affections, he can remember none to which he is hereditarily predisposed, as his father, his mother, his grandfather and grandmother on both sides, were long-lived and very healthy people.

About the 3rd of July last, he was suddenly seized with severe palpitation of the heart; his face and lips became very livid, and he had extreme vertigo—yet there was no aberration of speech, and if other symptoms were present, they were not detected. He was confined to his bed from this attack for two weeks, after which he was able to be up and about attending to his daily avocations, and in the course of a few days his health seemed quite restored, and he felt as well as ever. He remained in this condition, however, only a short time, for, on the 14th of August, as he was crossing over to New York City on the ferry-boat, he was paralyzed on the right side, and lost the power of speech. At the time of the attack he was standing on the deck, engaged in reading the newspaper and whistling a tune, when suddenly, and without the least warning, the lines became blurred, and his tune was cut short as though he had been shot. He fell instantly to the deck, and was unconscious for a few minutes, but he soon regained his senses, was carried home, and a physician was summoned, who on examination found that he was paralyzed on the right side of his body. The leg and arm were both involved, the face was drawn to the left side, and his speech was so much impaired that he was totally unable to utter a single audible sound. The condition of the pupils, of the muscles of the eyes, and of the tongue, were not observed; neither was the tactile sensibility tested, nor the mental symptoms, if any were present, noted. He now had a vein opened in his arm, yet it was some time before the blood would flow; but after it commenced he was bled profusely, whereupon he immediately rallied, spoke a few words, and then walked up stairs without assistance. In the course of a week the paralysis of the face had entirely disappeared, and he had quite recovered the normal use of his arm and leg; but in regard to his speech he was not so fortunate, for he was only able to make use of three or four words, —namely, "one" "two," and "yes, mam,"—which he used indiscriminately in reply to all questions. From this period until the 14th of October last, when he came under the observation of Dr. Wm. A. Hammond and myself at the New York State Hospital for Diseases of the Nervous System, he had improved very little in the acquisition of words, as will be seen from his condition at that time, which was as follows:

The dynamometer shows that there is the normal disparity between the right and the left hand, the patient being right-handed. There is no observable deviation of the face to the left side, even when its muscles are thrown into action through the influence of the emotions. The motility of the leg is at present unimpaired; the toe of the right foot does not catch or drag in walking, and the patient appears to use it fully as well as the other. The tongue, however, does not come out perfectly straight, as there is a slight deviation toward the left side; nevertheless there is no restraint in its movements, for the patient can move it in all possible directions with the utmost ease. He can at present whistle very well, which shows that he has nearly regained the power of co-ordinating the muscles of the lips, for after the facial paralysis had disappeared, and the mouth was restored to its normal position, he was even then unable to perform the movements with the lips which are required to act in harmony in order to produce a musical sound, so as to whistle an air. The tactile sensibility and the sensation of pain are normal on the whole of the right side of the body. He has no headache or other head symptoms at present; all his special senses are normal; the pupils are equal in size, and respond readily to light; he has no ptosis, no strabismus, or other impairment of vision. The ophthalmoscope discloses that the vessels of the retina and optic disk of the left eye are larger, more tortuous, and more numerous than those of the right, and the choroid of a redder hue. On examining the heart it was found to be enlarged, and the normal valvular sounds were much weakened. There was also a distinct intermission in the pulsations of the heart, which was also very irregular in its action. This intermission is not only perceptible in the precordial region, but also in the radial artery in each wrist. The most careful attention, however, failed to discover any murmur; the lungs were perfectly healthy; his bowels are regular, his urine natural, and his general health excellent; his appetite is good, he sleeps well, and if he could give expression to his thoughts, it seems as though he would be pronounced a tolerably healthy man.

He was the subject of a clinical lecture by Prof. Hammond, to the class of the Bellevue Hospital Medical College, and has regularly made his appearance in the lecture room every Saturday, appearing to take great interest in the remarks made on his case.

The vocabulary of this patient is at present restricted to a few such words as "one," "two," and "yes, mam," which expressions he makes use of in reply to nearly all questions, whether he intends to convey a negative or an affirmative answer, and between these he makes no choice, but uses either indiscriminately. He seems to comprehend perfectly everything that is said to him, yet it is a very difficult matter in a case like the present, where the patient is able to speak, to write, and to gesticulate only to a very limited extent, to form an accurate idea of his intellectual capacity. On asking him his name, he looked attentively at me and replied, "one two." "But that is not your name." He replied, "Yes, mam." "How old are you?" He smiled, closed all the fingers of both hands three times, and then held up two fingers. I answered, "Thirty-two." He said, "Yes, mam," nodded his head in approval, and then suddenly said, "That's so." I asked him to repeat the expression he had just used, and he replied, "One two." Say "one two." He answered, "Yes, mam." "Can you count?" He replied, "Yes, mam." "Say one." He answered correctly. "Say two." He repeated the number. "Say three." He paused, and then suddenly said, "Too much." This was about the extent of this man's vocabulary. I now tried to make him repeat a word after me, but all my efforts were in vain, if I except the numerals one and two. He can say "one two," but he cannot say "two one." Although he is unable to give expression to his thoughts, he is nevertheless quite intelligent. He does a large and lucrative business, which he superintends in person, and his agent and wife assure me that they can see no change in his capability of managing his own affairs. He knows the use of all objects with which he was formerly familiar, although he cannot call them by name. If he be asked the name of an object—as a book, for instance—he will make the sign of denial until the correct name is mentioned, and then he will smile approvingly and say, "Yes, mam." I have tried him very often in this manner, and he has always been correct, when the object was such as he ought to be familiar with. On asking him if he could read, he immediately signified to me that he could; then, running to my table, he took up a newspaper, and, apparently following the lines, repeated the most outlandish gibberish. His wife, who was present, told me he often read the papers, and that she was quite certain that he understood many of the subjects therein contained. As I rather doubted this, I very carefully tested his capacity in this respect, and I was not surprised to find that not only was he unable to read and to distinguish one word from another, but that he was equally unable to point out a single numeral or a single letter of the alphabet correctly. Again, as he persisted that he understood perfectly, I conversed on several subjects, and requested him to tell me which one he was reading. When I spoke of any theme of every-day conversation, he pointed to the article in the paper and exclaimed, "Yes, mam," "That's it;" and he was always wrong. He can write only a few words, such as Brooklyn, Jersey City, and his own name, without a copy; and although he writes these names distinctly and legibly, yet he is totally unable to pronounce them, even if the words be plainly enunciated, each separately, for him. He can point out correctly only those words which he is able to write without a copy, and these are only two or three in number, as I have already mentioned. With a copy before him, however, he can write as well and as fluently as he ever could in his life. He is perfectly conversant with time, locality, and circumstance. He is not only expert in managing his extensive business and providing for all the wants of his family, but in handling money he is both proficient and correct. Although he cannot point out a single numeral correctly, yet he can tell the time; and in taking his history he constantly pulled out his almanac, and referred me to the dates, which his wife subsequently informed me were right. He is at times very irritable and excitable. His wrath is easily aroused, and all his emotions are more or less exalted. The repetition of the same word, and the characteristic sign of impatience after an unsuccessful attempt to pronounce a word, are not as well marked in this case as many others which have fallen under my observation; nevertheless they are present, and are at times quite conspicuous.

Often while conversing with this patient, when he found that he was unable to make me understand what he wished to communicate, would he rush to my table in an excited manner, seize a pencil, and strive to write the words which he could not express, but after much effort he would only succeed in writing his own name, Brooklyn, or Jersey City, as if these words were stereotyped in his mind; and then his countenance would show plainly the grief he felt at his utter failure, and he would walk back to his seat, exclaiming, in a doleful tone, "Too much, too much."

Again, this patient seems to have no idea of gesticulation or pantomime, and excepting the employment of his fingers in expressing numbers, he uses them to a very limited extent, and only then when all other means of making his ideas intelligible have failed. In enumerating by means of his fingers, I have never known him to make a mistake. All attempts, however, to make him repeat any letter of the alphabet have so far proved fruitless.

The ease with which laughter or crying is excited in Capt. D. is a prominent point in his case, as it is very seldom met with in aphasic patients. The words that he articulates are clear and distinct; there is no stammering or tendency to clip the words. If asked to repeat the numeral five, he will say, "one two," and hold up five fingers, which shows conclusively that he understands, and that there is no difficulty in the organ of hearing.

The treatment adopted in this case was the passage of the primary galvanic current through the head, one pole being placed on the right mastoid process and the other on the left; then changing the poles, by placing one on the nape of the neck and the other on the forehead. These applications were made every other day, and in addition the patient has taken a sixteenth of a grain of strychnine three times a day internally, together with five drops of the phosphorated oil in emulsion, with mucilage, thrice daily also.

After he had been under treatment about a month he seemed to improve, for he acquired several new words, which he employed when he was engaged in superintending his men at work. Suddenly he would exclaim, "Come on now," "That's all," "How do you know?" "Yes, sir," and the like. The words are only made use of on special occasions, and he cannot be made to repeat them, although he has just given utterance to them. On the 10th of November, while asleep in bed, he awoke suddenly, and immediately felt extreme vertigo to such a degree that he was obliged to lie down again, and at the same time violent convulsive movements took place in his right hand. There was no loss of consciousness during the attack, and no aberration of speech manifest after it. On examining him a few days afterwards, the only change in his condition that I could discover was that he seemed much less intelligent than he did on former occasions. Since this attack I have learned from his wife that he very often suffers from dizzy spells, but that he has never lost consciousness. Yet these speedily pass away, and they leave behind them no sequelæ. The 17th of November last he commenced to have very severe headaches, which would come on at one o'clock in the afternoon and continue until five. After lasting a few days he informed me of the fact, and I discontinued the use of the strychnia. Since then the vertigo and the headaches have entirely disappeared.

Thanksgiving-day he was disappointed because a lady whom he had invited was not present at dinner; and on the day after, she called at his house to express her regrets, when he suddenly cried out, "Why did you not come to dinner?" This was enunciated as distinctly as you or I could have said it.

Gradually Captain D. is acquiring new words and phrases, but these he does not employ on common occasions; and he still adheres to "one two" and "yes, mam," for ordinary conversation. He has lately shown some knowledge of numbers, and on testing him in this respect, I found he was able to add up as many as three columns of figures. He is also able to write many numerals, and his wife says he can calculate his profits very readily at present by writing them down on paper and adding them up. His figures are not always distinct, but notwithstanding this, some of them are very well formed. By testing him on this point I found that his wife's assertion was correct. He has lately formed the habit of swearing very profanely, and he makes use of several well-known oaths with the utmost facility. In this respect at least, his powers of co-ordination are good. He is aware that he swears, and seems to take a certain degree of pleasure in so doing. He is also very fond of notoriety. He has had his picture printed on his circulars, which he takes great pride in distributing broadcast over the country, and to the medical class of the college. He has undoubtedly improved since he came under our observation in his mental processes; but so far as his speech is concerned, he still seems to adhere with great pertinacity to "one two" and "yes, mam;" nevertheless, the phrases which he utters from time to time show an increased activity on the part of the brain, and consequently we have every reason to hope for continued improvement. The following are the prominent points of interest in this case:

1. The disease came on suddenly, and from the history, could be attributed to no other cause than embolism.
2. The paralysis was on the right side of the body, while the lesion was on the left side of the brain.
3. The aphasia was a concomitant of the right hemiplegia, which fact demonstrates, almost to a certainty, the site of the embolus in the left middle cerebral artery.
4. There is a forgetfulness of words, or an impairment of the idea of language, which is called amnesic aphasia. This form exists in this case, but is by no means so prominent as the other, as will be seen from the history. If there were no trouble in coordinating the muscles concerned in articulation, the patient would experience no difficulty in repeating the word after it had been distinctly pronounced for him, provided the interval of time between the word spoken and the attempt to enunciate it were not too long. On testing Captain D. in this respect, I found him very deficient.
5. There is an impairment of the expression of language, or ataxic aphasia. This is the marked form in the present case. It depends upon an inability to will the movements necessary to produce articulate language.
6. There is agraphia, which is of the amnesic variety. In this case he does not remember written forms, but when a copy is before him, he writes fluently.
7. While the ataxia of speech is the most conspicuous, the agraphia is limited almost entirely to the amnesic variety.
8. There is an impairment of gesture or pantomime, which is particularly marked in this case.

